# An Unexpected Case of Generalized Tetanus

**DOI:** 10.7759/cureus.75326

**Published:** 2024-12-08

**Authors:** Laura Baptista, Lorrane Viana, Catarina Almeida, Inês S Pinheiro, Rafaela Araújo

**Affiliations:** 1 Internal Medicine, Unidade Local de Saúde da Região de Aveiro, Aveiro, PRT; 2 Infectious Diseases, Unidade Local de Saúde da Região de Aveiro, Aveiro, PRT; 3 Immunohemotherapy Service, Unidade Local de Saúde da Região de Aveiro, Aveiro, PRT; 4 Medicine, Unidade Local de Saúde da Região de Aveiro, Aveiro, PRT; 5 Intensive Care Unit, Unidade Local de Saúde da Região de Aveiro, Aveiro, PRT

**Keywords:** delayed diagnosis, developed countries, emergency critical care, generalized tetanus, multiple departments, post-exposure prophylaxis, tetanus, tetanus immunoglobulin, vaccination

## Abstract

Tetanus is a disease of the nervous system caused by a toxin produced by *Clostridium tetani*, an anaerobe found in high concentrations in the soil. The occurrence of tetanus is related to contaminated traumatic wounds, and most patients have had some failure in their immunization. However, there are rare case reports of generalized tetanus in patients with proper vaccination schemes who failed to receive appropriate prophylaxis after high-risk exposure. A 79-year-old woman presented to the emergency room (ER) with a large wound on her leg caused by an iron pipe during agricultural work. Tetanus immunization status was confirmed (the last booster received seven years prior to the injury), the wound was debrided and sutured, and she was discharged. Four days later, she returned to the ER due to high fever, neck pain, and inability to completely open her mouth. Hypertension, cervical stiffness, and sardonic smile were observed and soon evolved into severe dysautonomia, which required prolonged sedation and analgesia. Since there was a strong suspicion of tetanus, tetanus immunoglobulin (TIG) and tetanus vaccine (TTV) were administered, new surgical wound debridement was performed, and intravenous antibiotic therapy with metronidazole and ceftriaxone was initiated. She would later be discharged to a continued care facility where, despite a slow recovery, she progressed favorably. Tetanus is a disease that can present with different clinical forms and severity, but it is relatively easy to prevent when appropriate pre- and/or post-exposure prophylaxis is carried out. It is of utmost importance that physicians remain up to date in the latest scientific knowledge and guidelines surrounding tetanus, so as to avoid lapses in the administration of TTV when indicated. Such was the case with our patient: given that her last tetanus TTV booster was administered more than five years prior to the high-risk injury she sustained, prophylaxis with TTV should have been promptly administered during the initial ER episode. The rarity of tetanus in developed countries should not overshadow the gravity of the disease and the potential for severe outcomes if left untreated.

## Introduction

Tetanus is an infectious disease of the nervous system caused by the toxin-producing anaerobe *Clostridium tetani*, which is found in the soil and gastrointestinal tracts of humans and other animals [1]. The occurrence of tetanus is therefore intricately linked to traumatic wounds contaminated with pathogenic microorganisms. While the prevalence of tetanus has significantly diminished (the age-standardized incidence rate of tetanus decreased from 10.26 per 100,000 in 1990 to 1.03 per 100,000 in 2019 [2]), it persists as a formidable risk for individuals lacking adequate vaccination, particularly in regions characterized by limited healthcare resources and infrastructure, such as the developing world [2].

Since *C. tetani *spores cannot be eliminated from the environment, complete eradication is unattainable. Consequently, prioritizing immunization and meticulous wound care is imperative for effective tetanus prevention. In affluent nations with abundant resources, the annual incidence of tetanus is on a diminishing trajectory, primarily attributable to widespread vaccination initiatives [3]. Despite this positive trend, a substantial proportion of adults remain inadequately vaccinated against tetanus. A noteworthy challenge is observed in cases where individuals lack a comprehensive series of tetanus toxoid immunizations and receive suboptimal prophylaxis following a traumatic injury. Intriguingly, there have been instances of tetanus occurrence in individuals presenting pre-existing antitetanus antibodies, highlighting the complexity of the host-pathogen interaction in tetanus pathogenesis [4].

Upon introduction through a breach in the skin, dormant *C. tetani *spores undergo germination into bacilli under anaerobic conditions. These bacilli, in turn, generate the potent endotoxin tetanospasmin. This neurotoxin acts by impeding the presynaptic release of neurotransmitters that normally serve to inhibit muscular contraction. Consequently, this interference results in uncontrolled muscle contractions, giving rise to clinical spasms [5]. The intricate mechanism of tetanospasmin's action underscores the profound impact of *C. tetani *on neuromuscular physiology, elucidating the pathophysiological basis of tetanus manifestations.

Due to the aversion of *C. tetani *to grow in healthy tissues, a specific set of conditions must occur to facilitate the production of tetanospasmin within the human host. This synergistic combination typically entails the absence of protective antibodies, in conjunction with at least two or more predisposing factors. These factors encompass a penetrating injury leading to the introduction of *C. tetani* spores, potential co-infection with other bacteria, the presence of devitalized tissue, the existence of a foreign body at the site, and/or localized ischemia [6]. *C. tetani* can gain entry into the human body through chronic wounds, such as pressure ulcers, complications arising from diabetes [7], and dental cavities [8]. In these instances, the protracted nature of the wounds may create an environment conducive to the germination of dormant spores and subsequent bacillary growth. This emphasizes the importance of recognizing diverse entry points and the varied contexts in which *C. tetani* may establish infection, thereby broadening our understanding of the potential pathways for tetanus acquisition.

The median incubation period for tetanus is typically eight days, with a range extending from three to 21 days [6], a duration notably shorter in neonatal tetanus. Notably, a shorter duration between symptom onset and the appearance of spasms correlates with increased disease severity [9]. This temporal relationship underscores the urgency of prompt recognition and intervention in the clinical management of tetanus.

Tetanus presents in four distinct clinical forms, namely, neonatal, localized, cephalic, and generalized, with the latter being the most prevalent and severe [5].

In generalized tetanus, which constitutes the majority of cases, trismus is the predominant initial symptom in over 80% of patients. Early phases of generalized tetanus may exhibit symptoms of autonomic overactivity, including sweating and tachycardia, progressing to later stages characterized by profuse sweating, arrhythmias, labile arterial blood pressure, and fever. Characteristic features of generalized tetanus include tonic contraction of skeletal muscles and intermittent, intensely painful muscular spasms, a stiff neck, opisthotonos, sardonic smile, rigid abdomen, periods of apnea, upper airway obstruction, and dysphagia [10].

Localized tetanus is characterized by tonic or spastic muscle contractions in a specific extremity or body region, with the potential to progress to generalized tetanus. Cephalic tetanus primarily involves the cranial nerves, manifesting initially as dysphagia, trismus, and focal cranial neuropathies. The facial nerve is most commonly affected. This unique presentation can sometimes lead to a misdiagnosis of stroke prior to the appearance of typical features of generalized tetanus [11].

Neonatal tetanus arises due to inadequate aseptic practices in managing the umbilical stump of offspring born to mothers with insufficient immunization. Typically occurring five to seven days post-birth, neonatal tetanus underscores the critical importance of maternal immunization and proper perinatal care to prevent this form of the disease [12].

The diagnosis of tetanus is clinical and does not require laboratory confirmation, as culture can lead to both false-positive and false-negatives, and it is therefore based on a compatible clinical presentation in the absence of a more likely cause. Therefore, it is especially important to remain vigilant even in less obvious clinical circumstances. Maintaining awareness in low-incidence settings, such as developed countries where the occurrence of tetanus is less frequent, is crucial for prompt recognition and intervention that can change the outcome of this potentially fatal condition [13].

## Case presentation

**Figure 1:**
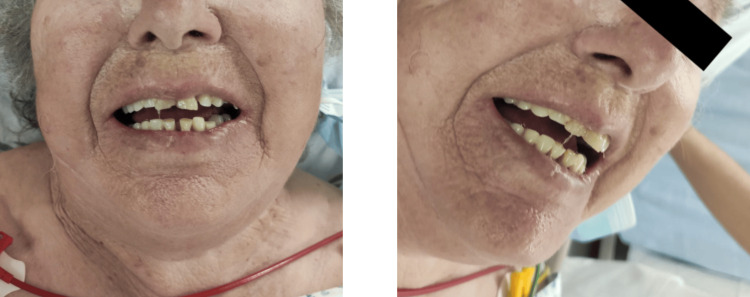
Sardonic smile exhibited by the patient on her second ER visit. *Risus sardonicus*, or sardonic smile, is a highly characteristic, abnormal, sustained spasm of the facial muscles that appears to produce grinning caused by tetanus (among other causes).

**Figure 2:**
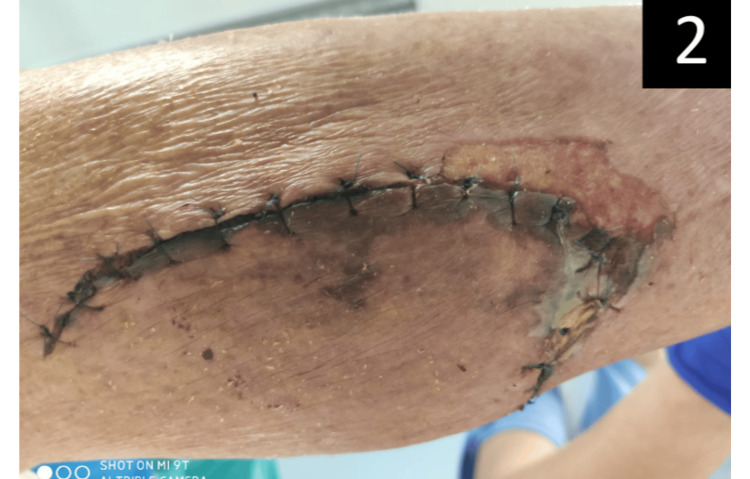
Wound presenting exhuberant inflammatory signs during the patient's second visit to the emergency room (ER).

The authors present the case of a 79-year-old woman, previously independent with unremarkable medical history. The patient sought attention in the emergency room (ER) in May 2021 after sustaining a substantial leg wound resulting from an injury involving an iron pipe during agricultural activities. Prompt medical intervention occurred within six hours of the incident, involving wound cleaning and suturing. The patient's tetanus immunization status was diligently assessed, revealing a history of at least three doses, with the last administration in 2014. Subsequently, she was discharged with a prescription for amoxicillin/clavulanate. Four days later, the patient returned to the ER, reporting fever, neck pain, and an inability to open her mouth. Clinical examination revealed afebrile status, hypertension, tachycardia, and cervical and submandibular stiffness, along with the presence of a sardonic smile (Figure 1)*, *raising suspicion of tetanus. The wound now exhibited pronounced inflammatory signs (Figure 2).

A single intramuscular dose of 500 units of human tetanus immunoglobulin (HTIG) was administered around the wound, accompanied by a 0.5 mL dose of tetanus toxoid-containing vaccine (TTV) in the left upper arm. Intravenous antibiotic therapy, consisting of metronidazole and ceftriaxone (the latter added as the mixed infection was suspected at the time), was initiated and sustained for 10 days.

Laboratory results showed elevated inflammatory markers, including leukocytosis (14.000 x 10^9^/L) with neutrophilia and elevated reactive C-protein of 14.92mg/dL. Soon thereafter, the patient presented trismus and important cervical stiffness that hindered adequate airway management and required orotracheal intubation, profound sedation, curarization, and mechanical ventilation, and she was admitted to the intensive care unit (ICU).

**Figure 3:**
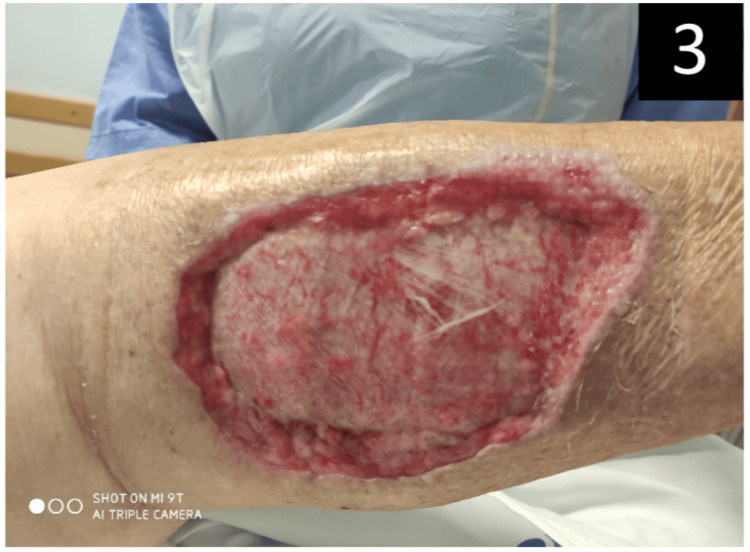
Patient's wound after various episodes of surgical debridement.

During the patient’s 56-day stay in the ICU, she presented severe dysautonomia, with labile yet severe hypertension episodes, with the need for magnesium sulfate, deep sedation, labetalol perfusion, and concomitant oscillating noradrenaline doses. Intrathecal baclofen was administered for muscle spasm control and later changed to dantrolene, as sedation was weaned off. Multiple surgical wound debridements were performed during the patient's hospitalization (Figure 3), and early tracheotomy was performed due to the likelihood of prolonged mechanical ventilation.

Following this critical period, the patient was transferred to the Infectious Diseases ward where, despite a slow recovery, she exhibited favorable progress. Ultimately, she was discharged to a continuous care unit, marking a positive outcome in her protracted and intricate clinical course.

## Discussion

Diagnosis and clinical management

The confirmation of tetanus diagnosis poses a challenge, as it is a clinical syndrome characterized by a lack of specific laboratory tests for confirmation. Anti-tetanus antibodies are typically undetectable in the majority of patients with tetanus, and reports indicate that the disease may still occur in individuals with antibody concentrations surpassing the commonly cited protective threshold of 0.01 IU/L [14]. While laboratory tests cannot definitively confirm tetanus, they serve a crucial role in excluding conditions that might mimic its symptoms, such as strychnine poisoning, neuroleptic malignant syndrome, and stiff-person syndrome [15]. 

In our case, the diagnosis was presumptive and based on a clinical evaluation guided by the patient's medical history. Strychnine poisoning (while possible as the patient dealt with farm work and, presumably, had contact with strychnine-containing rodenticide) was deemed less likely than tetanus, given the history of a recent penetrating injury and inadequate immunization status. Stiff-person syndrome was unlikely as well, given the insufficient response to benzodiazepines (which is a hallmark of stiff-person syndrome), or the absence of an auto-immune background.

Specific tetanus laboratory tests were not conducted during the patient's admission. While such tests could have provided valuable data for scientific analysis, it is emphasized that their absence did not impact the patient's management. The diagnosis and subsequent treatment were founded on the clinical presentation, highlighting the importance of clinical acumen in the absence of specific confirmatory laboratory tests.

Upon her second presentation to the ER, a presumptive diagnosis of generalized tetanus was promptly established, leading to immediate and comprehensive therapeutic interventions. The patient received TTV (0.5 mL) and human tetanus immunoglobulin (HTIG) (500 units) intramuscularly, administered at different locations, with a preference for HTIG around the wound. Concurrently, wound management involved a series of surgical debridements to eliminate spores and necrotic tissue. Antimicrobial therapy, comprising metronidazole targeting *C. tetani* and ceftriaxone due to suspected mixed wound infection, was initiated to address potential bacterial co-pathogens. The multidimensional strategy implemented during the second ER episode aimed not only to neutralize circulating toxins but also to eradicate the source of infection and mitigate potential complications, marking a crucial step towards the patient's recovery.

The optimal management of tetanus involves treatment in an ICU, primarily for effective control of tetanus complications, with a focus on early and aggressive airway management, as was the case for our patient. The overarching goals of treatment encompassed halting toxin production, neutralizing unbound toxins, controlling muscle spasms, managing dysautonomia, and providing general supportive care [16,17].

Wound debridement is considered a crucial component of the therapeutic approach, aiming to eliminate spores and necrotic tissue that may harbor the causative agent, so the patient underwent several surgical wound debridements throughout her stay.

Benzodiazepines, a conventional pharmacotherapeutic modality, represent a customary and generally efficacious approach to the mitigation of rigidity and spasmodic manifestations in tetanus patients. It is noteworthy that individuals afflicted with tetanus often exhibit an atypically elevated tolerance to the sedative properties of benzodiazepines, persisting in an alert state even when administered at doses conventionally expected to induce anesthesia. Concomitant propofol infusion has demonstrated effectiveness as an additional agent in the management of spasms in this clinical context through deep sedation and anesthesia [15]. However, much as it happens in generalized tetanus, sedation alone proved insufficient, with neuromuscular blocking drugs coming into play, with continued infusion required for sustained effects. Muscle spasms were challenging to manage as curarization and sedation were weaned off, with favorable results when simultaneously administering magnesium sulfate (maintaining therapeutic levels between 2 and 4 mmol/L) and intrathecal baclofen, later switched to dantrolene, to help reduce the need for ventilation and allowing the sedation to be progressively tapered off [16].

Blood pressure control was often suboptimal due to autonomic hyperactivity, presenting with oscillating and labile blood pressure values: either presenting with severe hypertension requiring adrenergic blockade (labetalol (0.25 to 1 mg/min) is favored for its dual alpha and beta-blocking properties), but also hypotensive values as deep sedation and curarization were needed. Magnesium sulfate infusion helped reduce the need for other drugs to control muscle spasms, as a calcium antagonist that induces vasodilation and presynaptic neuromuscular blockages, preventing catecholamine release from the presynaptic neurons [18].

Due to the likelihood of prolonged mechanical ventilation, early tracheostomy is often indicated in tetanus cases, and our patient was not an exception.

The patient's manifestation of a short incubation period contributed to increased disease, further underscored by the atypical duration of her tetanus presentation, which exceeded the typical four to six weeks. Notably, our patient's hospitalization spanned 72 days, with a substantial portion spent in the intensive care unit. The prolonged course of her illness aligns with the potential for extended recovery periods in certain tetanus cases, in particular when coupled with post-intensive care syndrome (PICS), as was the case with our patient.

In concert with supportive care, airway management, autonomic instability, and muscle spasm control, this multifaceted therapeutic strategy aimed to improve patient outcomes and minimize the complications associated with tetanus, particularly in our case, where the initial prophylactic measures were not optimally administered.

Tetanus survivors commonly experience profound psychological challenges arising from the impact of the disease and its treatment. The arduous nature of the illness, often requiring prolonged hospitalization, intensive care, and intricate medical interventions, contributes to a spectrum of psychological issues that can significantly affect the mental well-being of individuals who have endured tetanus. These challenges may include but are not limited to post-traumatic stress, anxiety, depression, and adjustment disorders, underscoring the importance of comprehensive support and mental health care for tetanus survivors as part of their overall recovery process.

Nevertheless, despite the prolonged hospitalization, severe disease, and advanced age, the patient in question is currently faring well, with excellent results after a comprehensive rehabilitation program. With the aid of a cane, she continues dealing with farmwork and is presently mostly independent in her daily life activities.

Prevention and broader implications

The retrospective analysis of our patient's medical course reveals a lapse in the adequacy of the initial ER treatment and post-exposure prevention. Our patient first presented to the ER with a high-risk injury for tetanus. High-risk wounds include penetrating wounds, wounds containing dirt/soil/feces/saliva, or wounds containing devitalized tissue [9]. 

Despite having a complete tetanus immunization primary series (at least three doses), the patient had received her last TTV booster seven years prior to the injury.

Given that her last tetanus TTV booster was administered more than five years before the injury, in accordance with prevailing guidelines [13,19] summarized in Table 1, prophylaxis with TTV should have been promptly administered during the initial ER episode. This proactive measure might have potentially averted her subsequent progression to generalized tetanus.

**Table 1:** Recommended post-exposure immunization regimens Unimmunized^a^: unknown vaccine history, unvaccinated people, or incomplete tetanus vaccine primary series HTIG: human tetanus immune globulin

	Low-risk wound	High-risk wound
Unimmunized^a^ patient	Vaccine recommended	Vaccine + HTIG recommended
Immunized patient	Vaccine recommended if last tetanus vaccine > 10 years ago	Vaccine recommended if last tetanus vaccine > 5 years ago

In developed nations, comprehensive vaccination programs have been instrumental in ensuring widespread coverage against tetanus. This includes routine immunization schedules and booster doses, creating a population with enhanced immunity to the causative agent, *C. tetani*. In addition, the prompt administration of TTV and/or HTIG following injuries, especially those with a high risk of tetanus, has proven to be a crucial preventive measure. This approach neutralizes circulating tetanus toxins and provides passive immunity, offering immediate protection while the individual's immune system mounts a response.

Individuals with a history of immunosuppression, diabetes, and intravenous drug use may also be at an elevated risk for tetanus. Notably, age assumes a significant role in this context, with the incidence of tetanus and the associated risk of mortality being notably higher in individuals aged 60 years and above [20]. It is pertinent to acknowledge that case-fatality rates for non-neonatal tetanus in resource-limited countries exhibit a wide range, spanning from 5% to 50%. However, the majority of tetanus patients, particularly in settings with modern supportive care, demonstrate favorable recovery trajectories despite the severity of the condition. The notable reduction in tetanus incidence over recent decades, particularly in developed countries in well-resourced healthcare settings, can be attributed significantly to the implementation of robust vaccination practices and the timely administration of TTV and/or HTIG following injuries [2].

The preeminent risk factor for tetanus is unequivocally the absence of appropriate vaccination. It is imperative that adequate risk assessment be made at the time of a patient’s contact with health providers after exposure, so as to avoid progression of the disease.

## Conclusions

Diagnosing tetanus in developed countries can be challenging, given its diminishing prevalence and the consequent waning awareness among healthcare professionals. However, the right clinical setting should consistently prompt consideration of tetanus, and in such instances, physicians play a pivotal role in the timely recognition and accurate diagnosis of this disease. It becomes imperative for physicians to stay informed and updated with the latest scientific knowledge and guidelines surrounding tetanus, so as to avoid lapses in the administration of TTV when indicated, such as was the case with our patient.

The rarity of tetanus in developed countries should not overshadow the gravity of the disease and the potential for severe outcomes if left untreated. The medical community's commitment to remaining vigilant, well-informed, and adherent to preventive protocols is fundamental in safeguarding public health and mitigating the potential impact of tetanus.
